# A central mechanism enhances pain perception of noxious thermal stimulus changes

**DOI:** 10.1038/s41598-017-04009-9

**Published:** 2017-06-20

**Authors:** B. Petre, P. Tetreault, V. A. Mathur, M. W. Schurgin, J. Y. Chiao, L. Huang, A. V. Apkarian

**Affiliations:** 1 Department of Physiology, Northwestern University, 303 E Chicago Ave, Chicago, Ill, 60610 United States; 2 Department of Psychology, Northwestern University, 303 E Chicago Ave, Chicago, Ill, 60610 United States; 3 Anesthesia and PM&R, Northwestern University, 303 E Chicago Ave, Chicago, Ill, 60610 United States

## Abstract

Pain perception temporarily exaggerates abrupt thermal stimulus changes revealing a mechanism for nociceptive temporal contrast enhancement (TCE). Although the mechanism is unknown, a non-linear model with perceptual feedback accurately simulates the phenomenon. Here we test if a mechanism in the central nervous system underlies thermal TCE. Our model successfully predicted an optimal stimulus, incorporating a transient temperature offset (step-up/step-down), with maximal TCE, resulting in psychophysically verified large decrements in pain response (“offset-analgesia”; mean analgesia: 85%, *n* = 20 subjects). Next, this stimulus was delivered using two thermodes, one delivering the longer duration baseline temperature pulse and the other superimposing a short higher temperature pulse. The two stimuli were applied simultaneously either near or far on the same arm, or on opposite arms. Spatial separation across multiple peripheral receptive fields ensures the composite stimulus timecourse is first reconstituted in the central nervous system. Following ipsilateral stimulus cessation on the high temperature thermode, but before cessation of the low temperature stimulus properties of TCE were observed both for individual subjects and in group-mean responses. This demonstrates a central integration mechanism is sufficient to evoke painful thermal TCE, an essential step in transforming transient afferent nociceptive signals into a stable pain perception.

## Introduction

Attention and behavioral response to important stimulus features is the culmination of a cascade of filtering and amplification mechanisms in the nervous system. In the early stages of vision for instance edges are enhanced first by retinal ganglion cells and again by simple cells of the primary visual cortex^1, 2^, and in audition temporal and context filtering facilitates stimulus binding for recognition of complex sounds like species-specific vocalizations^3–6^. The understanding of analogous processes in pain perception is comparatively primitive, especially for processing of time varying stimuli. Rapidly changing step-wise thermal stimuli applied to the skin are perceptually salient due to disproportionate but transient change in pain intensity. Although initially observed as the familiar but disproportionate relief following stimulus decrements (“offset analgesia”)^7, 8^ temporal contrast enhancement (TCE) is more general and also occurs after noxious stimulus increments^9^.

Pain promotes defensive action and future avoidance behavior, which requires associating defensive behavior with resultant changes in pain^10^. A complete characterization of this process must incorporate an understanding of stimulus response dynamics, and in particular how stimulus changes are detected and interpreted. A temporal contrast enhancement mechanism must retain a memory trace of stimulus history and alter perceived stimulus intensity, and can occur at many levels of the neuraxis, whether as a consequence of adaptation in peripheral neurons, or more complex mechanisms in the central nervous system (CNS).

Several unsuccessful attempts have been made to link TCE to established CNS mechanisms of spatial and context dependent nociceptive filtering. Context dependent nociceptive filters like placebo analgesia operate through an opioidergic circuit located in the periaqueductal gray matter (PAG)^11^. While fMRI studies show the offset analgesia TCE phenomenon also correlates with metabolic activity in the PAG^12, 13^ opiate agonists and antagonists fail to alter TCE dynamics^14^. Likewise, spatial contrast enhancement, in the form of conditioned pain modulation (CPM), has been dissociated from offset analgesia by ketamine antagonism of NMDA receptors, which selectively inhibits CPM^15^.

Nevertheless, there are reasons to suspect some other CNS mechanism underlies nociceptive TCE. Across modalities contrast enhancement constructs behaviorally relevant perceptual features late in sensory processing, allowing for signal convergence and modulation by feedback projections^16, 17^, and, in the temporal domain, for longer information retention^6, 18, 19^. Additionally, asymmetric spatial interactions have been observed between time varying painful stimuli and simultaneous but remote noxious stimulation^7^. This at least reveals events in the CNS can interfere with pain dynamics because peripheral nociceptive neurons have receptive fields on the order of centimeters and are unaffected by remote stimulus changes^20, 21^. Consequently, we hypothesize a central mechanism underlies nociceptive TCE.

Responses to step-wise noxious stimuli cannot be reduced to a linear combination, or superposition, of responses to constituent boxcar stimuli, so this TCE is a nonlinear response. Here we leverage this property to probe for a central mechanism using psychophysics, theoretical modeling, and painful thermal stimuli. An optimal step-wise low-high-low stimulus paradigm is identified by exploring a recurrent feedback model trained on a preliminary dataset^22^. The complex stimulus is then fractured in two: a short high intensity boxcar stimulus bounded by a longer low intensity boxcar stimulus. Delivered simultaneously, their composite reproduces the low-high-low stimulus profile. A nonlinear response to the low-high-low stimulus is confirmed by comparison with responses to individual boxcars. We then bypass the ability of primary afferent fibers to detect changes in the overall stimulus profile by simultaneously delivering each boxcar stimulus to remote locations using two thermodes, and test if the response matches a superposition of constituent stimulus responses or is nonlinear. Nonlinear integration across peripheral receptive fields is sufficient to demonstrate a central mechanism for nociceptive TCE.

## Results

### Model exploration and stimulus design

**Figure 1:**
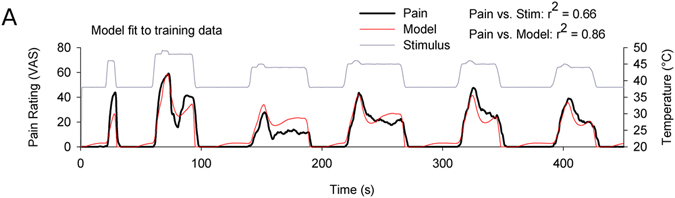
A nonlinear feedback model (red) captures mean response of 11 subjects (black) to a painful thermal stimulus sequence (gray). Stimuli are similar to those previously used to study “offset analgesia” and include a 1 °C × 5 s up/down step. Pearson correlations between pain vs. stimulus and pain vs. model prediction are shown.

A nonlinear stimulus-response function with perceptual feedback was fit to evoked pain ratings averaged across 11 naïve subjects (Eq. 1, Fig. 1A). The model fit performs well in capturing pain response features, sharing a greater proportion of variance with VAS ratings than does the stimulus (pain vs. stimulus: *r* ^2^ = 0.656; pain vs. model fit: *r* ^2^ = 0.855; forced timeseries data is not independent or identically distributed, but Pearson correlations serve as a similarity measure). The fit also shows a prominent nonlinearity following stimulus offsets which resolves within 15 s, matching the pain response during the first low-high-low stimulus epoch, but not later epochs, consistent with published reports on offset analgesia^7^. Model fitting was performed blind to model exploration or subsequent testing.

**Figure 2:**
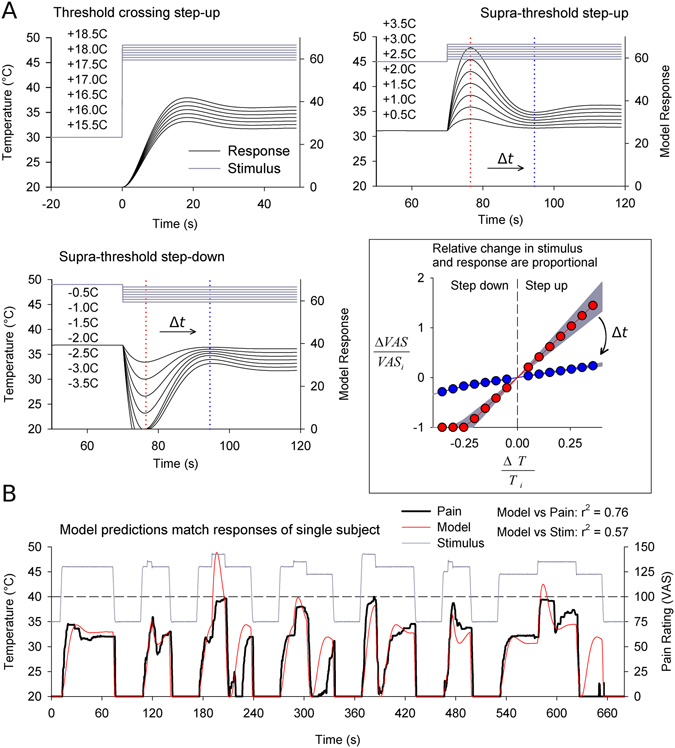
Model exploration suggests ideal stimulus parameters to resolve painful TCE. (**A**) The model step-response was explored outside the training space, and three different types of step responses were identified. Responses to threshold crossing steps peak at ~15 s while steps from supra-threshold ‘holding’ stimuli (*T*_*i*_) peak after ~7.5 s. Oscillations follow which stabilize after 45 s. Finally, positive and negative supra-threshold steps show symmetric responses. For supra-threshold steps, relative change in pain is proportional to relative change in stimulus with a time and *T*_*i*_ dependent proportionality constant (insert; color coordinated slopes are plotted for two time points marked on supra-threshold step response plots; shadows illustrate *T*_*i*_ ∈ [45 °C, 49 °C], *T*_*i*_ = 47 °C featured). Response dynamics are approximately invariant with *T*_*i*_ and representative step-response functions are shown. (**B**) Model predictions were qualitatively evaluated in a single subject using a variety of threshold crossing and supra-threshold steps. Results confirm presence of nonlinear damped oscillations in all three types of step-responses, confirms dependence of nonlinearity amplitude on step size, and that 2.5 °C steps transiently abolish pain (epochs 3–5, 7).

Model exploration was used to design a boxcar stimulus and a low-high-low complex stimulus which would evoke prominent and distinct nonlinearities in pain response. To find a candidate boxcar stimulus we first examined step functions that crossed the pain threshold (θ; initial temperature, *T*_i_; final temperature, *T*_f_; *T*_i_ < θ < *T*_f_; Fig. 2A, top left). Peak response was predicted 14–15 s after stimulus onset regardless of *T*_i_ and with magnitude proportional to *T*_f_. Time to peak and subsequent adaptation dynamics were independent of *T*_f_. For the complex stimulus we examined positive and negative steps taken from suprathreshold holding stimuli (θ < *T*_i_ < *T*_f_ and θ < *T*_f_ < *T*_i_). These also showed approximately temperature invariant dynamics. They were distinguished by a shorter 3–4 s time to peak (or trough), followed by prominent damped oscillations which stabilized within about 40 s after stimulus onset (Fig. 2A, top right, bottom left).

In typical fashion^23^ predicted ratios of pain intensity were proportional to ratios of stimulus intensity, but with a time and *T*_*i*_ dependent proportionality constant (Fig. 2A, insert). In other words,

1 $$\frac{pain({T}_{f})}{pain({T}_{i})}\propto \frac{{T}_{f}-{\rm{\theta }}}{{T}_{i}-{\rm{\theta }}}$$

In the range of experimentally tractable stimulus intensities (*T*_*i*_ ∈ [45 °C, 49 °C]), the dependence on *T*_*i*_ is trivial (Fig. 2A, insert, shaded gray area). Thus, as a practical consequence of this relationship, an approximately 25% decrease in temperature relative to threshold (~2.5 °C for 45–49 °C stimuli) was predicted to transiently abolish pain. These predictions were unambiguously confirmed by preliminary testing in one subject (Pearson correlation, VAS vs. stimulus: r^2^ = 0.570; VAS vs. model: r^2^ = 0.755; Fig. 2A).

**Figure 3:**
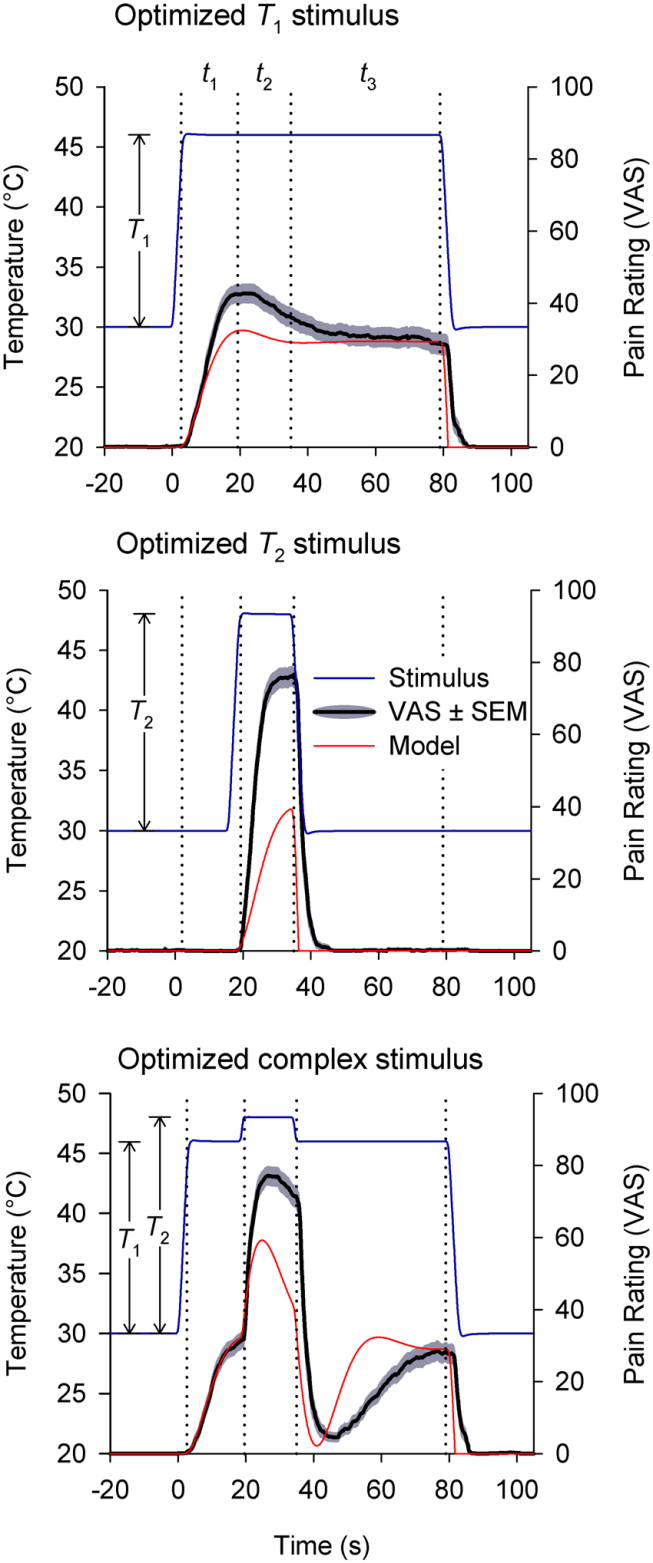
Group mean acute pain dynamics (black) from simple and complex single thermode stimuli (gray) are predicted accurately by the model (red). Three stimuli are shown (one per panel), but while stimuli are linear combinations of each other, the resulting pain ratings are not. Relevant temporal periods (t_1_-t_2_-t_3_) are labeled consistently across stimuli (15 s–15 s–45 s). Nonlinear pain dynamics are best resolved in the complex stimulus during t_3_ where a disproportionate transient pain reduction follows a small stimulus offset (“offset analgesia”). An additional more subtle nonlinearity is visible during t_2_ where a small increase in stimulus intensity results in a disproportionately fast rise in pain intensity. These properties cannot be explained through linear combinations of simple stimulus response relationships but are predicted by the model. (*T*_2_ = *T*_1_ + 2 °C, *N* = 20).

We applied these lessons in the design of a new low-high-low complex stimulus (15 s–15 s–45 s at *T*_1_-*T*_2_-*T*_1_, *T*_1_ > θ, *T*_2_ = *T*_1_ + 2 °C) and also designed corresponding decompositions of this stimulus into two constituent boxcars (15 s–15 s–45 s at either *T*_1_-*T*_1_-*T*_1_ or baseline-*T*_2_-baseline, hereon ‘*T*_1_ stimulus’ and ‘*T*_2_ stimulus’, respectively, baseline ≪θ; Fig. 3). 15 s stimulation periods ensure peak response is achieved, the final 45 s period resolves the full time course of perceptual enhancement and 2 °C offsets (96% predicted pain reduction) avoid the ceiling effects in VAS ratings seen during preliminary testing of the larger 2.5 °C steps (Fig. 2B).

### Complex stimulus paradigm maximizes nonlinear response

Nonlinear dynamics were tested using boxcar and complex stimuli in a novel group of 20 subjects. Results are consistent with model predictions (Fig. 3, Table 1). For boxcar stimuli, mean *T*_1_ stimulus response showed a time to peak of 15.3 s (bootstrapped CI_.95_ = [11.5, 18.3], *n* = 50000 samples; times taken from start of response which lags the stimulus), and the *T*_2_ stimulus response also follow this trajectory only truncated by the shorter stimulus duration (time to peak pain: 12.7 s, CI_.95_ = [9.3, 13.2]). The complex stimulus is a linear combination of *T*_1_ and *T*_2_ stimuli, but the mean pain response is not a linear combination of evoked responses to boxcar stimuli. Although *T*_2_ stimulation occurs on top of a preexisting *T*_1_ stimulation, the *T*_2_ evoked response features a shorter time to peak pain (t_2_ maximum: 5.4 s, CI_.95_ = [5.0, 8.2]) than occurs with the *T*_2_ boxcar stimulus (bootstrap *p* < 0.001). The model predicts this kind of TCE for supra-threshold step responses (3.6 s for supra-threshold vs. 14.6 s for threshold crossing steps). Additionally, the complex stimulus intensity returns to *T*_1_ after the 2 °C stimulus offset, at which point another prominent nonlinearity is visible in the form of an “offset analgesia” type transient downward deflection (t_3_ minimum predicted at 4.5 s; observed at 9.8 s, CI_.95_ = [6.6, 10.8]; predicted amplitude: 0.96, observed amplitude: 0.85 ± 0.067, mean ± SE; VAS amplitude calculated at t_3_ minimum with respect to mean of t_1_ max and t_3_ max). This demonstrates the intrinsic contrast enhancing nonlinearities predicted by the model can be captured in the group level pain responses.

**Table 1:** Timing of local extrema in group mean responses with bootstrapped 95% confidence intervals (bracketed).

	T_1_ alone	T_2_ alone	Complex	Near *T*_1_ + *T*_2_	Far *T*_1_ + *T*_2_	Opposite arm *T*_1_ + *T*_2_
*t*_1_ max	15.3 [11.5, 18.3]	—	—	—	—	—
*t*_2_ max	—	12.7 [9.3, 13.2]	5.4 [5.0, 8.2]	11.5 [9.6, 12.9]	11.7 [7.0, 13.4]	10.1 [7.2, 10.9]
*t*_3_ min	—	—	9.8 [6.6, 10.8]	11.5 [7, 15.6]	13.3 [4.7, 16.8]	19.5 [8.5, 36.0]
*t*_3_ max	—	—	40.1 [39.8, 44.2]	42.6 [31.3, 44.1]	37.5 [32.8, 44.1]	28.8 [11.0, 43.7]

### Subject level responses to dual thermode stimuli are nonlinear

**Figure 4:**
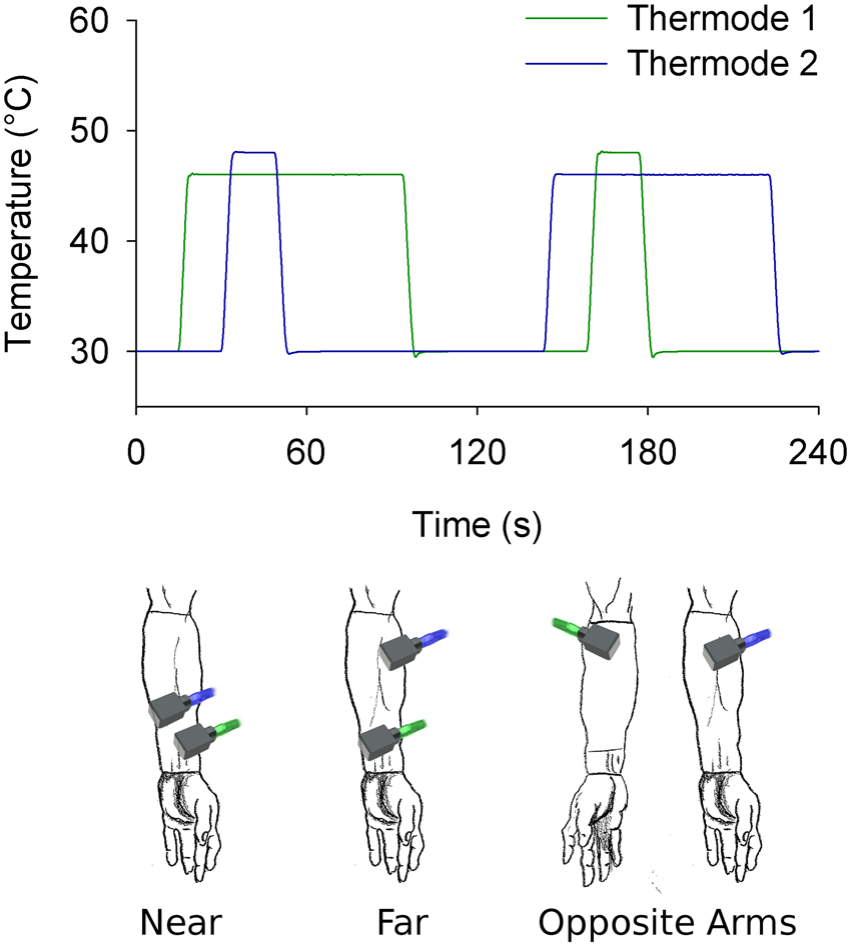
Dual thermode experimental design allows for simultaneous delivery of stimuli to different sites. A complex stimulus is deconstructed into its component features, which are then delivered on the ventral forearm either near one another, far from one another or contralaterally. Illustrated arms are a partial reproduction of Leonardo da Vinci’s Vitruvian man^35^.

Simultaneous dual thermode stimulation tested if central mechanisms are sufficient to evoke nonlinear contrast enhancing (TCE) pain dynamics. Only simple boxcar stimuli were used, but stimulus epochs featured coincident delivery of the *T*_1_ and *T*_2_ stimuli at different sites (Fig. 4). By design *T*_1_ stimulation began 15 s before -and ended 45 s after- *T*_2_ stimulation. Thus, the superposition of these two stimuli reproduces the optimal timeseries of the single thermode complex stimulus (Figs 3 and 4). Critically, spatial separation between thermodes during dual thermode stimulation exceeded single peripheral receptive field sizes. In order to potentially disambiguate the level of the neuraxis at which the prospective CNS mechanism might act, stimuli were delivered at three distances: near and far on the same arm, or on opposite arms (Fig. 4).

**Figure 5:**
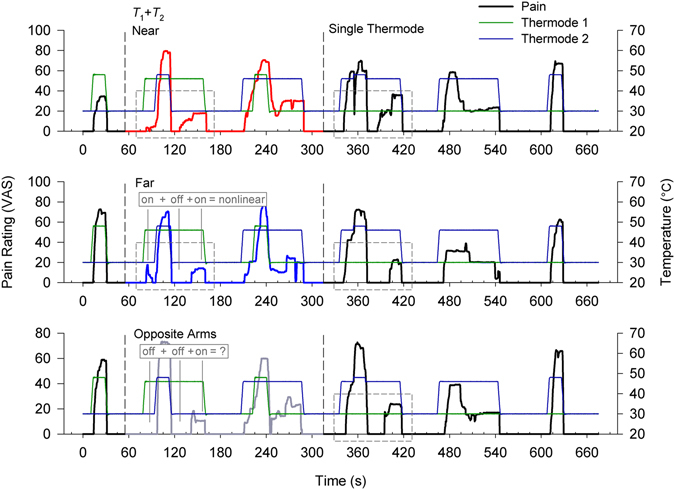
Stimulus and pain ratings from a representative subject exhibiting nonlinear pain for single complex stimuli and for dual stimuli configurations (near, far, and opposite arm). Each stimulation sequence consisted of two dual-thermode stimuli (red, blue, gray ratings), and three single thermode stimuli (black ratings). Dual thermode stimuli were delivered at three different distances from one another: near or far on the same arm or on opposite arms, and the order of these was randomized across subjects. The disproportionate diminution of perceived pain following a small offset shows that this subject rates all dual and single complex stimuli nonlinearly, in contrast to a linear sum of the simple stimuli. A more stringent but less ambiguous on-off-on criterion for nonlinear dynamics is also satisfied in many of these instances (gray boxes, one example and one counter example explicitly labeled): despite invariance of stimulus intensity there is pain before *T*_2_ stimulation, no pain immediately after *T*_2_ stimulation, and then pain comes back before the *T*_1_ stimulus ends. Thermode roles switch between the first and second dual thermode stimulus epochs.

In order to distinguish monotonic site specific sensitization and habituation from intrinsic nonlinear dynamics (TCE), responses were first evaluated according to a simple on-off-on criterion. At the single subject level, many responses reflect transient absence of pain during the final 45 s of stimulus epochs (e.g. Fig. 5). However, if a pain free period interrupts distinct periods of unambiguous *T*_1_ evoked pain, then neither habituation (a continuous decay process) nor sensitization (a continuous rise process) offers adequate explanation. Instead, a nonlinear TCE response provides the most parsimonious explanation (e.g. Fig. 5, gray dashed boxes).

This rudimentary on-off-on criterion for nonlinearity demonstrates the dual thermode stimuli evoke nonlinear dynamics which can be discerned within individual subject responses. The complex single thermode stimulus acts as a positive control, and evokes significantly more frequent nonlinear responses (69% non-linear, *n* = 52) than the simple *T*_1_ boxcar stimulus which in turn serves as a negative control for random VAS variance (4% non-linear, *n* = 55; ${{\rm{\chi }}}_{1}^{2}$ = 48.5, *p* < 1e-05; Cochran-Mantel-Haenszel Chi-squared test, accounting for stratification by stimulus intensity which was adjusted according to pain tolerance). The greatest incidence of nonlinearities among dual thermode stimuli occurs with delivery of both to the same forearm, either near each other or far apart (50% non-linear, *n* = 32, ${{\rm{\chi }}}_{1}^{2}$ = 26.3, *p* < 1e-05; 48% non-linear, *n* = 31, ${{\rm{\chi }}}_{1}^{2}$ = 21.5, *p* < 1e-05; resp). Delivery of *T*_2_ on the opposite arm shows less frequent nonlinear responses, but still more than from the single thermode *T*_1_ stimulus (19% nonlinear, ${{\rm{\chi }}}_{1}^{2}$ = 4.44, *p* = 0.035, *n* = 27; all tests taken with respect to the negative control and significant after Holm-Sidak correction for multiple comparisons; Table 2). Thus, dual thermode stimuli can produce nonlinear stimulus response dynamics, clearly discernable at the level of individual subjects, although there may be a distance or laterality dependence.

**Table 2:** Incidence of nonlinear responses with stratification by stimulus level (*T*_1_ = [44.5 °C, 45 °C, 46 °C, 47 °C]).

	Complex	Near *T*_1_ + *T*_2_	Far *T*_1_ + *T*_2_	Opposite arm *T*_1_ + *T*_2_	*T*_1_ alone
Trials with transient *VAS* = 0	36 [0,9,13,14]	16 [0,5,7,4]	15 [0,5,5,5]	5 [1,2,2,0]	2 [0,2,0,0]
*VAS* > 0 trials	16 [3,8,0,5]	16 [2,3,1,10]	16 [1,5,3,7]	23 [0,6,4,13]	53 [3,17,13,20]
Rate of nonlinear pain abolition	0.69**	0.50**	0.48**	0.18*	0.04

### Group level dual thermode responses match the nonlinear complex stimulus response dynamics

Dual thermode stimuli evoke nonlinear responses, but single thermode complex stimuli show rich and predictable dynamics not captured by the simple on-off-on criterion. For instance, the psychophysical model exploration and single thermode stimuli highlight characteristic maxima and minima of group mean TCE responses (Figs 2 and 3), while individual subject responses are discontinuous, resembling a random jump process with indistinct maxima and minima (e.g. Fig. 5), obscuring this precise temporal information. Employing group mean responses thus allows for further comparison between the precise dynamics of different stimulus responses.

**Figure 6:**
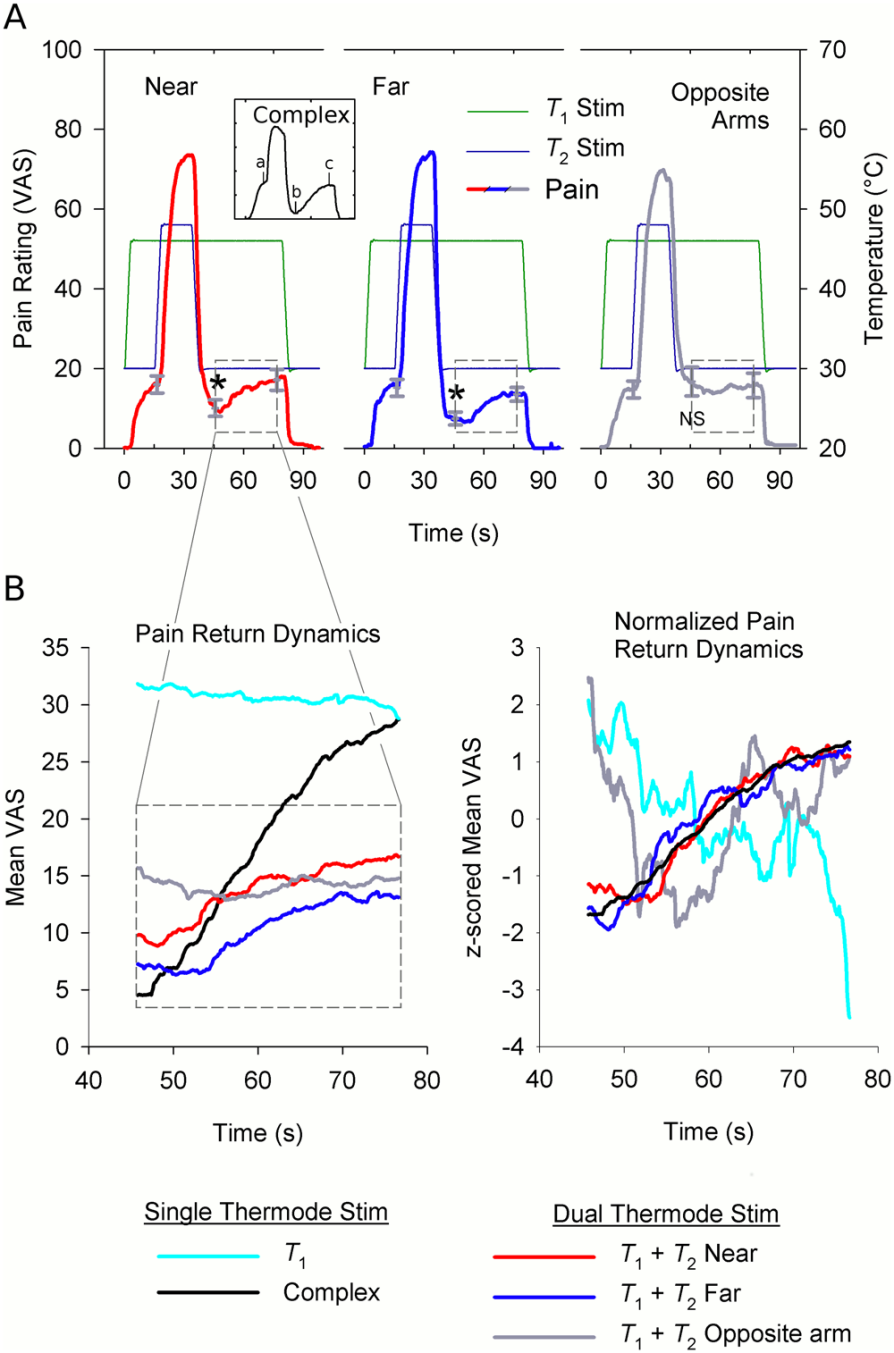
Near and far dual thermode stimuli produce a nonlinear response with the same dynamics as a single complex stimulus. (**A**) Simultaneous *T*_1_ stimulation and near or far *T*_2_ stimulation, but not *T*_2_ stimulation on the opposite arm, produces a nonlinear group-averaged pain response. Comparisons were performed based on timepoints selected from the complex stimulus response (insert): (a) maximal *t*_1_ VAS, (b) minimal *t*_3_ VAS and (c) maximal *t*_3_ VAS. Stimulus intensity was invariant between (a), (b) and (c) in dual thermode stimuli, but pain responses at (a) and (c) were significantly higher than (b) after near (*p* = 0.010) or far *T*_2_ stimulation (*p* = 0.002; planned contrast). No differences were found after *T*_2_ stimulation of the opposing arm (*p* = 0.87). (**B**) In near and far dual thermode stimuli the nonlinear response dynamics are the same as for the single thermode complex stimulus. Pain return dynamics were specifically examined between (b) and (c) (top panel, insert). Group averaged responses to near or far dual thermode stimuli significantly correlate with responses to the complex but not *T*_1_ stimulus while responses to opposite arm stimulation correlates with neither (left). Correlations between responses to near, far, and single thermode complex stimulation support the conclusion that the same process underlies all three nonlinear responses (multiple regression with ARIMA errors; near vs. complex: *t* = 4.54, *p* < 1e-05; far vs complex *t* = 1.99, *p* = 0.047; df = 437). Dynamical equivalence is best illustrated with variance normalized (z-scored) timeseries (right). *p < 0.05. NS = non significant.

Using group mean responses, time of (a) *t*_1_ peak pain, (b) *t*_3_ minimum pain, and (c) *t*_3_ peak pain were identified for the complex stimulus, and used as reference points for evaluating TCE in dual thermode stimulus responses (Fig. 6A). Differences in stimulus intensity between subjects did not affect reported pain or moderate TCE (p > 0.4 for T_1_ and TCE*T_1_ interactions, Table 3), so these terms were dropped from the model. However, the TCE reference points show significantly different pain ratings in near dual thermode stimuli, with pain at (a) and (c) significantly higher than (b) (planned comparison mean (a, c) – (b): 7.86 ± 2.75 VAS difference, mean ± SE., 47% pain reduction, *p* = 0.01). The same pattern occurs with far dual thermode stimuli responses (planned comparison: 7.15 ± 1.99 VAS difference, 50% pain reduction, *p* = 0.002), but not for opposite arm dual thermode stimuli (planned comparison: −0.39 ± 2.29 difference, 2% pain increase, *p* = 0.87; Table 4). Similarly, timing of *t*_3_ extrema observed with near and far stimuli compare favorably with extrema of the single thermode complex stimulus (bootstrapped contrast of local minima timing, near vs. complex: *p* = 0.24, far vs. complex *p* = 0.18, opposite vs. complex: *p* < 0.001; Table 1). Therefore, during a stimulus of invariant intensity, nonlinear dynamics are evoked across near and far dual thermode stimuli at the same timepoints which characterize the single thermode complex stimulus response.

**Table 3:** Preliminary mixed model of dual stimulus responses, measured at timepoints of complex stimulus extrema, shows there is no interaction between TCE and stimulus intensity between subjects.

	Coef.	SE	df	*t*-stat	p			
*Near* (*preliminary*)						F	df	p
Intercept	14.09	2.10	17	6.71	3.64e-06*			
TCE_ac-b_	7.86	2.83	17	2.78	0.0128*	4.20	2,17	0.0329^NS^
TCE_a-c_	−1.72	2.72	17	−0.63	0.537			
S (demeaned)	6.42	2.21	17	2.90	9.93e-3*			
*T*_1_ (demeaned)	0.403	2.34	17	0.17	0.865			
TCE_ac-b_ *T_1_	−0.16	3.16	17	−0.05	0.960	0.30	2,17	0.745
TCE_a-c_ *T_1_	2.13	3.04	17	0.70	0.493			
S*T_1_	−7.70	2.47	17	−3.11	6.27e-03*			
*Far* (*preliminary*)						F	df	p
Intercept	11.81	1.29	16.28	9.17	7.83e-08*			
TCE_ac-b_	7.16	2.04	17.72	3.52	2.52e-03*	6.69	2,17.95	6.75e-03*
TCE_a-c_	2.42	3.38	16.14	0.72	0.48			
S (demeaned)	5.75	1.89	15.87	3.05	7.68e-03*			
*T*_1_ (demeaned)	−0.79	1.40	14.40	−0.56	0.58			
TCE_ac-b_ *T_1_	−0.39	2.22	17.84	−0.18	0.86	0.02	2,18.06	0.98
TCE_a-c_ *T_1_	−0.03	3.68	16.23	−0.02	0.98			
S*T_1_	0.03	2.06	16.01	0.013	0.99			
*Opposite arms* (*preliminary*)						F	df	P
Intercept	14.76	3.00	16.28	4.90	1.56e-04			
TCE_ac-b_	−0.50	2.35	17.83	−0.21	0.834	0.07	2,16.22	0.94
TCE_a-c_	−1.15	3.42	15.08	−0.34	0.740			
S (demeaned)	4.59	3.30	16.74	1.39	0.183			
*T*_1_ (demeaned)	2.23	2.58	14.62	0.87	0.401			
TCE_ac-b_ *T_1_	−0.83	2.59	18.64	−0.32	0.751	0.05	2,16.95	0.88
TCE_a-c_ *T_1_	−0.39	3.76	15.68	−0.10	0.919			
S*T_1_	−0.97	2.94	15.30	−0.33	0.745

**Table 4:** Final mixed effects model shows a statistically significant nonlinear response for near and far dual thermode stimuli, but not the opposite arm stimulus.

	Coef.	SE	df	*t*-stat	p			
*Near* (*final*)						F	df	p
Intercept	14.09	2.09	17.31	6.75	3.11e-06*			
TCE_ac-b_	7.86	2.75	18	2.86	0.0104*	4.44	2,18	0.0271^NS^
TCE_a-c_	−1.72	2.68	18	−0.64	0.531			
S (demeaned)	6.42	2.21	17.02	2.90	9.90e-3*			
*T*_1_ (demeaned)	0.30	2.12	17	0.14	0.888			
S*T_1_	−7.44	2.45	17	−3.04	7.35e-03*			
*Far* (*final*)						F	df	p
Intercept	11.81	1.26	17.35	9.35	3.44e-08*			
TCE_ac-b_	7.15	1.99	19.00	3.59	1.95e-03*	6.94	2,19.2	5.39e-03*
TCE_a-c_	2.42	3.28	17.17	0.74	0.477			
S (demeaned)	5.79	1.82	16.98	3.17	5.55e-03*			
*Opposites* (*final*)						F	df	p
Intercept	14.62	3.09	17.19	4.74	1.86e-04*			
TCE_ac-b_	−0.39	2.29	19.20	−1.7	0.865	0.06	2,17.61	0.946
TCE_a-c_	−1.08	3.34	16.02	−0.32	0.750			
S (demeaned)	2.40	2.49	16.49	0.97	0.349			

Although local maxima and minima reflect characteristic stimulus dynamics and edge enhancement, trajectories between extrema also characterize pain dynamics. However, most group mean stimulus responses were too abrupt to robustly model. Changes in pain in response to stimulus changes affect the statistical properties of the VAS timeseries (e.g. mean and variance) in a complicated way. These changes effectively introduce heterogeneity in the probability distribution of the timeseries from one stimulation period to the next (i.e. timeseries structure is “non-stationary”). This problem is compounded by the relatively short duration between stimulus changes (e.g. t_1_ and t_2_ pain ratings only span 214 frames each), which limits the number of effective degrees of freedom available to model these statistical properties. This confounds linear timeseries modelling of complete stimulus responses. Nevertheless, the pain return during *t*_3_ is sustained during a period of constant stimulation, which simplifies modelling. Dual thermode responses between timepoints (b) and (c) were therefore modeled as a linear combination of two independent variables, each representing a competing hypothesis: (1) the single thermode complex stimulus response (nonlinear hypothesis) and (2) the single thermode *T*_1_ stimulus response (linear hypothesis). Although competing, these hypotheses are not necessarily mutually exclusive, and combining both together in a single model recognizes the possibility that responses to dual thermode stimuli may be a hybrid of single thermode stimulus-response patterns.

Regression models of near and far dual thermode responses support the nonlinear hypothesis (multiple regression with ARIMA(1, 1, 0) errors; near *β*_complex_ = 0.167, *t* = 4.54, *p* < 1e-05; far *β*_complex_ = 0.083, *t* = 2.03, *p* = 0.043; *df* = 437). The psychophysical model predicts the overall shape of the nonlinear trajectory should be the same regardless of how much pain is reported (i.e. dynamics show amplitude invariance) (Fig. 2A), a property clearly illustrated when comparing amplitude (i.e. variance) normalized responses to the complex stimulus with responses to ipsilateral dual stimuli. Conversely, neither linear nor nonlinear hypothesis offered a consistent explanation when modeling responses to the opposite arm stimulus, suggesting it may have unique dynamical properties (Fig. 6B, Table 5).

**Table 5:** Regression models of dual thermode stimulus responses during t_3_ as a linear combination of single thermode stimulus responses.

	Coef.	SE	*t*-stat	P-value
*Near*				
AR(1)	0.570	0.033	17.500	—
β Complex Resp.	0.167	0.037	4.537	7.38e-06*
β *T*_1_ Resp.	0.061	0.061	1.009	0.314
Variance	0.002	0.000	22.739	—
*Far*				
AR(1)	0.388	0.037	10.393	—
β Complex Resp.	0.083	0.042	1.999	0.047*
β *T*_1_ Resp.	0.041	0.067	0.614	0.5395
Variance	0.002	0.000	22.007	—
*Opposite arms*				
AR(1)	0.373	0.038	9.692	—
β Complex Resp.	−0.038	0.038	−0.994	0.321
β *T*_1_ Resp.	−0.003	0.066	−0.046	0.963
Variance	0.002	0.000	19.009	—

## Discussion

Temporal contrast enhancement in pain has thus far mainly been explored in the context of “offset analgesia” and a single stimulus pattern. Here we formally generalize this process to an entire class of stimuli with a nonlinear recurrent feedback model of temporal integration, and identify an invariance relationship underlying responses to step-wise dynamic stimuli. This enables simple and accurate construction of a highly effective novel stimulus paradigm for studying TCE in pain. Using simultaneous stimulation of different skin sites, we show the CNS is sufficient to perform nonlinear *spatiotemporal* integration of pain, and that resulting TCE pain dynamics are shared with nonlinear *temporal* integration of single stimuli.

Model extrapolations predict three distinct nonlinear stimulus-response motifs, two of which are contrast enhancing, and all are confirmed by single thermode stimuli. TCE responses to incremental stimulus increases and decreases are symmetric, peak around five seconds after stimulus onset and rapidly rebound. These dynamics enhance saliency of stimulus changes, and are absent in responses to initial stimulus onset. Critically, the stimulus features of the model training dataset are mainly 5 s long, too short to fully resolve stimulus-response motifs which show characteristic extrema and relaxation dynamics over 15–45 s of constant stimulation. Nevertheless, modeling successfully predicts the magnitude of downward deflecting nonlinearities, and peak and rebound dynamics of their upward deflecting counterparts. Although each of these motifs has been shown in some form before^8, 9^, this is the first time to our knowledge that they are systematically characterized and unified under a single theoretical framework. This shows that TCE pain dynamics behave as if there is an underlying recurrent feedback process.

The model’s ability to successfully extrapolate stimulus-responses outside of its training space lends credence to its use for mechanistic insight and as an interpretive lens, but limitations must also be recognized. Because our usage intent was primarily instrumental other models were not considered for comparison, but alternatives have been proposed which successfully predict response dynamics like adaptation and habituation^24^, processes our model was not designed to accommodate. Perhaps as a result our model overestimates the rate of pain rebound for the complex stimulus. Additionally, the predicted response symmetry between stimulus increases and decreases contradicts recent findings using painful laser stimuli^9^, which suggest more abrupt contrast enhancement for stimulus increments. However, it should be noted our model breaks symmetry in a similar manner as stimuli deviate from idealized steps, e.g. by using finite stimulus ramp rates. Any mechanistic insights gained from this model must undoubtedly be interpreted in the context of supplemental mechanisms affecting stimulus response properties. In fact, continuous rating of subjective states could be influenced by many factors such as attention, expectations, prior experience, and even trust in the experimenter. We imposed minimal control on such factors. Most importantly, the instructions of what to rate are critical, and subtle changes in the latter may lead to different outcomes. Finally, our model parameters were derived based on data collected mainly in males, while dual thermode experiments were conducted primarily in females. Although the robust extrapolation of model outcomes to the dual thermode results suggests that sex may not be an important contributor to pain TCE mechanisms, this remains to be systematically studied, as sex is an important factor in pain psychophysics. The small dynamical range of our thermodes limited the number of subjects that we could study; it remains unclear whether this introduced bias in our model fit. Further studies are needed to clarify the limits of the model’s extrapolations and dependence on stimulation apparatus and modality, task conditions and demographic composition.

The role of peripheral nociceptive neurons in TCE has not been systematically explored. Different classes of first order afferents contribute to stimulus dynamics by conveying unique stimulus information depending on response thresholds, latencies and adaptation dynamics. For instance, low threshold nociceptive C-fibers begin responding at 39–41 °C^25^, are unmyelinated and relatively slow to transmit afferent signals^26^ and rapidly adapt to stimulation^27^, properties conducive to detecting slow stimulus changes. Type-II Aδ fibers also rapidly adapt to stimulation but are much faster acting^28^ providing a second complementary method through which to detect fast stimulus changes^29^, while type-I Aδ fibers^27^ are slow adapting and might signal persistent stimulation. However, activity of first order afferents does not correlate with experimental tonic pain sensations^27^. Although we cannot *a priori* preclude that mechanisms in the periphery contribute to TCE dynamics, pain sensations appear more likely to be indirectly constructed from stimulation of the sensory epithelium, just as in vision, where a crisp and stable sensory representation is indirectly constructed from the radically different retinal image that is subject to constant saccadic jitter and optic distortions by ocular tissues and fluids.

Nevertheless, this study’s primary finding shows central mechanisms are sufficient for temporal contrast enhancement in thermal pain perception. When complex stimuli are deconstructed into constituent boxcars and delivered to the same arm either near or far apart, the subject’s response is not merely a superposition of responses to the constituent stimuli. Instead, a characteristic nonlinear, group mean response is observed with unambiguous subject level incidence. Receptive fields of first order nociceptive afferents are on the order of centimeters, while distances between thermodes exceed these and are up to an order of magnitude larger in the ‘far’ configuration. Therefore, subjects must integrate information from different peripheral afferent receptive fields to experience a TCE pain response. This entails nonlinear spatiotemporal integration of these stimuli at a higher level in the CNS.

Although we don’t distinguish between pain ratings and pain experience, there are undoubtedly differences due to reporting errors and the interpretive decisions inherent in self report. As we illustrate with single subject data, self report is discontinuous and is frequently a succession of abruptly changing steps, while (subjectively speaking) experience is continuous. Much of this error variance cancels out when averaging across subjects. We mitigate the risk of an interpretive confound by using an optimal stimulus paradigm. Identifying a nonlinear response depends on the temporal contrast of experienced pain, and our optimized stimulus paradigm ensures this contrast is maximized and highly salient, as shown by single thermode stimuli (85% pain decrease) and ipsilateral dual stimuli (50% pain decrease). This improves on all existing studies of offset analgesia since gross changes in pain intensity are much easier to identify and report than subtle ones.

Our experimental design precludes peripheral temporal integration of dual thermode stimuli, but the results do not provide further insight regarding the level of the neuraxis at which integration occurs. It may occur anywhere there are neurons with receptive fields large enough to circumscribe both thermodes, but candidates already exist at the level of the spinal dorsal horn (e.g. wide dynamic range neurons^30^). Thermodes were positioned on different sides of the radial midline of the arm, so it is likely different dermatomes were stimulated and spatial integration occurred across spinal segments. It was further hoped responses to opposite arm stimuli might identify a supraspinal mechanism with a bilateral receptive field. While the incidence of complete transient abolition of opposite arm pain remains statistically significant, the group dynamics during the prospective pain return period (t_3_) do not match the boxcar stimulus (as required by the superposition principle), the complex stimulus, nor a linear combination of the two. An additional nonlinear spatiotemporal integration process may yet be involved. Therefore, our findings do not distinguish between prospective segmental, intersegmental or supraspinal mechanisms.

Inhibition of pain after painful stimulation of a remote site might be achieved by spatial filtering acting in parallel with temporal contrast enhancement. For instance, in CPM one painful stimulus suppresses responses to additional painful stimuli elsewhere on the body. Although CPM and TCE in pain are mediated by distinct mechanisms^15^, dual thermode stimuli are both spatially and temporally integrated. However, transient pain reduction during our dual thermode stimuli follows the same dynamics as a single thermode complex stimulus, supporting the conclusion that nonlinear responses to dual thermode stimuli are the result of a mechanism shared with nonlinear responses to single thermode stimuli rather than any additional mechanism of spatial filtering.

The construction of sensory representation through successive levels of spatiotemporal filtering and amplification is a fundamental principle underlying the organization of the nervous system. By matching dynamics elicited by spatially localized or dispersed time-varying noxious stimuli we demonstrate the existence of a CNS mechanism for temporal contrast enhancement of thermal painful stimulus changes in pain perception.

## Methods

### Subjects

Data were collected from 20 individuals recruited from the Northwestern University community. All provided informed consent for all experiments, were right handed, at least 18 years old (age: 26 ± 3 years, mean ± SD; 6 males), and had no history of chronic pain, depression, dementia or other psychiatric conditions. A model training dataset was also obtained from 11 subjects recruited from the Chicago area (age: 23 ± 2 years, mean ± SD; 8 males). An additional 9 subjects were excluded from the training dataset due to equipment malfunction (3 subjects) or due to failure of a subset of training stimuli to evoke pain (6 subjects). All experiments were approved by and conducted in accordance with the ethical principles set forth by the Northwestern University institutional review board.

### Equipment

Stimuli were delivered by two Medoc PATHWAY ATS thermodes (30 × 30mm) controlled by two separate PCs, but were calibrated using a common external reference thermometer to protect against unintended stimulus mismatches. Temperature and event related data was sampled at 200 Hz. Real-time pain ratings were obtained using a finger device with VAS feedback, and sampled at 14.3 Hz using in house LabView software. Visual feedback was rendered as a moveable yellow bar alongside a number scale (0–100)^31^. The LabVIEW software also controlled a digital input/output device (National Instruments NI USB-6501) which delivered a 0.4 Hz heartbeat (synchronization) signal to each Medoc device during trial runs. These reference signals synchronously triggered stimulus delivery and facilitated alignment of stimuli and rating data during preprocessing.

Experiments were conducted in a purpose built space consisting of two adjacent rooms to minimize contact between experimenter and subject. The experimenter, PCs and Medoc systems were in one, while the subject, stimulating thermodes, feedback monitor and finger span device were in the other. Subjects were provided with and instructed in the use of an emergency stop button which would terminate stimuli if they became intolerable.

### Modeling

Pain was modeled using a nonlinear second order differential equation which has been previously described^22^.

2 $$\begin{array}{rcl}\ddot{p} & = & {\alpha }F({T},{\theta })-{\beta }\dot{{p}}+{\gamma }(\dot{{T}}({t})-{\lambda }){p}({t})\\ {F}({T},{\theta }) & = & \{\begin{array}{c}T-\theta ,\quad T\ge \theta \\ \quad 0,\quad T < \theta \end{array}\end{array}$$

The model was fit to a training dataset which was not used further in this study. Five stimuli were delivered consisting of 5s–5s–15s or a 5s–5s–30s stimulation, following a *T*_1_-*T*_2_-*T*_1_ temperature profile, where *T*_*1*_ ∈ [44 °C, 48 °C] and *T*_2_ = *T*_1_ + 1 °C. Stimulus timing profiles and 15 s or 45 s interstimulus intervals were pseudorandomly selected and preprogrammed into the stimulator, but delivered identically to all participants (Fig. 1). Mean stimulus-response data was used to obtain the model fit with a random walk over the parameter space. The obtained model fit was used throughout the rest of this study: (θ, α, β, γ, λ) = (37.1913, 2.4932, 36.7552, 0.0204, 0.0169).

### Experimental Task

Subjects were instructed to rate their overall pain from thermal stimulation using the finger device. The experimental task at times involved rating pain during delivery of two simultaneous stimuli of different intensity, but our *a priori* hypothesis only requires examining the periods flanking this period. Rather than introducing superfluous task demands by asking the subject to attend to a specific thermode, instructions were deliberately vague with respect to the source of the stimulus. Thus subjects were allowed to freely choose how to reconcile the two potentially competing stimuli in their rating.

Before experimental trials, subject pain thresholds and tolerance were evaluated. Six 10 s thermal stimuli were delivered at differing intensities to the right volar forearm. Stimuli differed in 1 °C increments, and each was used twice in random order. The procedure was repeated until (1) all stimuli used were painful but tolerated, (2) subject rated stimuli of equal intensity the same (±10 VAS units). Between repetitions stimuli were adjusted as needed to accommodate tolerances and pain thresholds of subjects. The least tolerant individuals completed training using 44 °C, 45 °C and 46 °C stimuli, while those with highest pain threshold completed training with 47 °C, 48 °C and 49 °C stimuli.

Experimental stimulation involved 2 noxious stimulus intensities (*T*_1_ and *T*_2_, where *T*_1_ + 2 °C = *T*_2_; baseline = *T*_1_ − 16 °C), used in 3 stimulation sequences, each with six stimulation epochs (Fig. 5). Overall, these thermal pulses were either a complex, low-high-low sequence as used in “offset-analgesia” studies, or its component low and high pulses, presented either on the same skin site, or at different skin locations. Sequences had identical stimulus programming: 15 s of *T*_2_, a dual thermode stimulus (75 s *T*_1_, 15 s *T*_2_), a second dual thermode stimulus (temperatures swapped), a 15s–15s–45s *T*_1_-*T*_2_-*T*_1_ complex stimulus, a 75 s *T*_1_ stimulus and finally another 15 s *T*_2_ stimulus. The dual thermode stimuli were timed such that their superposition would match the profile of the complex stimulus. Relative stimulus intensities were fixed, but absolute intensities were adjusted for pain thresholds and tolerances (*T*_1_ = [44.5 °C, 45 °C, 46 °C, 47 °C], N = [1, 5, 7, 7], resp.). Spatial configurations of thermodes (near, far or opposite arms, Fig. 4) were changed between sequences, and the order of sequences was randomized across subjects. Stimuli were delivered to the left ventral forearm, except for the opposite arm stimulus, which was delivered to a site on the right ventral forearm unaffected by the training task. 6 °C/s temperature ramp rates were used.

### Data analysis

#### Preprocessing

Matlab was used to resample thermode data to match the finger device sampling rate using heartbeat signals to determine appropriate timestamps. Stimulus-response lag was determined by the maximal cross correlation of stimulus and response for single thermode boxcar stimuli (average lag: 3.36 ± 1.6 s, mean ± SD). Finally, onset and offset times for responses to specific stimulus features of interest (i.e. *t*_1_, *t*_2_ and *t*_3_) were projected by discounting the temperature rise and fall rates and incorporating the average stimulus-response lag.

#### Hierarchical Linear Modelling

Inference on mean TCE was drawn using hierarchical linear models in order to control for between subject covariates (e.g. stimulus intensity) while also modelling variability within subject. Pain intensity (VAS) served as the dependent variable, measured at three timepoints per stimulus epoch (Fig. 6A, insert). Several considerations informed model selection. First, the study’s *a priori* hypothesis calls for a non-monotonic response characterized by two periods of high pain interrupted by a period of lower pain. This suggests a specific planned contrast (a and c vs. b in Fig. 6A). Second, the relationship between stimulus intensity and TCE (if any) is unknown and is modeled. Third, previous studies have shown site specific and nonspecific sensitization depend on stimulus intensity^24^, but neither affect magnitude of offset analgesia^32^ (a special case of TCE), therefore sensitization and its interaction with stimulus intensity, but not with TCE, are modeled. Finally, within subject stimulus intensity is constant across all timepoints of interest (a, b, c), therefore stimulus intensity and its higher order effects are only modeled at the group level. At the subject level the only random effects modeled are TCE and sensitization. The full model is summarized by the following equations,

3 $$\begin{array}{ccc}{\bf{V}}{\bf{A}}{\bf{S}} & = & {\bf{X}}{\bf{B}}{\boldsymbol{+}}{\bf{Z}}{\bf{u}}{\boldsymbol{+}}{\bf{e}}\\ {\bf{X}} & = & [{\bf{1}}{\boldsymbol{,}}{\bf{T}}{\bf{C}}{{\bf{E}}}_{{\bf{a}}{\bf{c}}{\boldsymbol{-}}{\bf{b}}}{\boldsymbol{,}}{\bf{T}}{\bf{C}}{{\bf{E}}}_{{\bf{a}}{\boldsymbol{-}}{\bf{c}}}{\boldsymbol{,}}{\bf{S}}{\boldsymbol{,}}{{\bf{T}}}_{{\bf{1}}}{\boldsymbol{,}}{{\bf{T}}}_{{\bf{1}}}\ast {\bf{S}}{\boldsymbol{]}}\\ {{\bf{Z}}}_{{\bf{i}}} & = & [{\bf{1}}{\boldsymbol{,}}{\bf{T}}{\bf{C}}{{\bf{E}}}_{{\bf{a}}{\bf{c}}{\boldsymbol{-}}{\bf{b}}}{\boldsymbol{,}}{\bf{T}}{\bf{C}}{{\bf{E}}}_{{\bf{a}}{\boldsymbol{-}}{\bf{c}}}{\boldsymbol{,}}{\bf{S}}{\boldsymbol{]}}\\ TC{E}_{ac-b}^{j} & = & \,\{\begin{array}{c}\frac{1}{3},\\ -\frac{2}{3},\end{array}\,\begin{array}{c}period(a)\,\,or\,\,(c)\\ period(b)\end{array}\\ TC{E}_{a-c}^{j} & =\, & \{\begin{array}{c}\frac{1}{2},\\ -\frac{1}{2},\end{array}\,\begin{array}{c}period(a)\\ period(c)\end{array}\end{array}$$

where **X** is the higher level design matrix, and **Z** is a block diagonal design matrix composed of **Z**_**i**_, the subject level design matrices. **S** is the stimulus number, since we delivered two, and models sensitization effects. TCE_ab-c_ is the planned contrast. The model was fit using restricted maximum likelihood estimation as implemented in the LME4 package in R. The lmerTest package was used for inference with Satterthwaite’s approximation for effective second level degrees of freedom. Because TCE is tested in three different thermode configurations (near, far and opposite arms), Holm-Sidak correction for multiple comparisons is employed to control the 0.05 false positive rate.

The higher level design matrix fits are reported in (Table 3). Although plausible *a priori*, many of these higher order parameter estimates are not statistically significant. Stepwise regression strategies would argue for their removal, but such methods can be problematic in hierarchical models due to the effect of higher level terms on lower level parameter estimates. Terms that are present at the first level of the model are not considered for removal due to the difficulty of correctly quantifying the effective reduction in model complexity, and such terms are also retained at the second level, but Bayes information criteria is used to determine which temperature terms to remove, since these are only present at the second level (Table 4). Nevertheless, because the correct approach to such model selection problems remains an open question in the statistical literature^33^, and different approaches might have justified a different final model selection we also present the preliminary models.

#### Dynamics of nonlinearity

Timeseries data was analyzed using an autoregressive integrated moving average (ARIMA) model^34^.

4 $${Y}={\boldsymbol{BX}}+{u}({t})$$

5 $$(1-\sum _{{i}=1}^{{p}}{{\phi }}_{{i}}{{L}}^{{i}}){(1-{L})}^{{\boldsymbol{d}}}{u}({t})=(1+\sum _{{i}=1}^{{q}}{{\theta }}_{{i}}{{L}}^{{i}}){{\varepsilon }}_{{t}}$$

ARIMA models capture linear relationships between points *within* timeseries (*u*(*t*)), producing a modified error term (*ε*) against which the statistical significance of parameter estimates *between* timeseries (**B**) can be evaluated (*L* ^*i*^, lag operator; *φ*_*i*_, autocorrelation weight of *u*(*t*) vs *u*(*t*-*i*); *θ*_*i*_, moving average weights).

To determine the error model order (*p*, *d*, *q*) we regress independent variables from the dependent variables and inspect the autocorrelation and partial autocorrelation plots of the residuals. These revealed a parsimonious solution with timeseries dominated by lag 1 autocorrelation at the level of the first difference and no moving average component, an ARIMA(1, 1, 0) model. Analysis of Bayesian information criteria for models with autoregressive and moving average terms within 2 orders of an ARIMA(1, 1, 0) model supports this conclusion. Thus all effect sizes for parameters of interest are evaluated while controlling for ARIMA(1, 1, 0) errors. This yields more conservative inferences than would result from a standard regression model.

### Data availability

Raw and preprocessed data used in this study are available from the corresponding author upon request.
